# Perturbations in the apoptotic pathway and mitochondrial network dynamics in peripheral blood mononuclear cells from bipolar disorder patients

**DOI:** 10.1038/tp.2017.83

**Published:** 2017-05-02

**Authors:** G Scaini, G R Fries, S S Valvassori, C P Zeni, G Zunta-Soares, M Berk, J C Soares, J Quevedo

**Affiliations:** 1 Translational Psychiatry Program, Department of Psychiatry and Behavioral Sciences, McGovern Medical School, The University of Texas Health Science Center at Houston, Houston, TX, USA; 2 Laboratory of Neurosciences, Graduate Program in Health Sciences, Health Sciences Unit, University of Southern Santa Catarina, Criciúma, Brazil; 3 Center of Excellence on Mood Disorders, Department of Psychiatry and Behavioral Sciences, McGovern Medical School, The University of Texas Health Science Center at Houston, Houston, TX, USA; 4 Deakin University, IMPACT Strategic Research Centre, School of Medicine, Faculty of Health, Geelong, VIC, Australia; 5 Orygen, The National Centre of Excellence in Youth Mental Health, Parkville, VIC, Australia; 6 The Centre for Youth Mental Health, Department of Psychiatry, Parkville, VIC, Australia; 7 Florey Institute for Neuroscience and Mental Health, the University of Melbourne, Parkville, VIC, Australia; 8 Neuroscience Graduate Program, The University of Texas Graduate School of Biomedical Sciences at Houston, Houston, TX, USA

## Abstract

Bipolar disorder (BD) is a severe psychiatric disorder characterized by phasic changes of mood and can be associated with progressive structural brain change and cognitive decline. The numbers and sizes of glia and neurons are reduced in several brain areas, suggesting the involvement of apoptosis in the pathophysiology of BD. Because the changes in mitochondrial dynamics are closely related with the early process of apoptosis and the specific processes of apoptosis and mitochondrial dynamics in BD have not been fully elucidated, we measured the apoptotic pathway and the expression of mitochondrial fission/fusion proteins from BD patients and healthy controls. We recruited 16 patients with BD type I and sixteen well-matched healthy controls and investigated protein levels of several pro-apoptotic and anti-apoptotic factors, as well as the expression of mitochondrial fission/fusion proteins in peripheral blood mononuclear cells (PBMCs). Our results showed that the levels of the anti-apoptotic proteins Bcl-xL, survivin and Bcl-xL/Bak dimer were significantly decreased, while active caspase-3 protein levels were significantly increased in PBMCs from BD patients. Moreover, we observed the downregulation of the mitochondrial fusion-related proteins Mfn2 and Opa1 and the upregulation of the fission protein Fis1 in PBMCs from BD patients, both in terms of gene expression and protein levels. We also showed a significantly decrease in the citrate synthase activity. Finally, we found a positive correlation between Mfn2 and Opa1 with mitochondrial content markers, as well as a negative correlation between mitochondrial fission/fusion proteins and apoptotic markers. Overall, data reported here are consistent with the working hypothesis that apoptosis may contribute to cellular dysfunction, brain volume loss and progressive cognitive in BD. Moreover, we show an important relationship between mitochondrial dynamics and the cell death pathway activation in BD patients, supporting the link between mitochondrial dysfunction and the pathophysiology of BD.

## Introduction

Bipolar disorder (BD) is a chronic, debilitating illness with a global prevalence of up to 4.8%, which is associated with medical and psychiatric comorbidity and increased standardized mortality ratios.^1, 2^ Acute episodes are associated with cognitive and functional impairments,^3^ which tend to worsen with the progression of the illness.^4, 5, 6, 7^ Despite superior cognitive capacity often present in at risk-individuals,^8^ individuals with a long-term course of illness present significant impairment in neuropsychological performance when compared to early-stage patients and controls.^4, 9^

Although several different mechanisms have been proposed for the pathogenesis of BD, its pathophysiology remains unknown.^10^ Numerous hypotheses have been postulated to underpin the neurobiology of BD, including the interaction of molecular, cellular and behavioral mechanisms with susceptibility genes, environmental stressors and biochemical mechanisms.^4^ Regional reductions in central nervous system volume, which are likely due to the atrophy and loss of neurons and glial cells, may be linked to an increase or imbalance in apoptosis,^11, 12, 13^ and it is known that mitochondria play a critical role in the regulation of apoptosis.^14^ Kim *et al.*^12^ reported a significant decrease in the levels of Bcl-2 and brain-derived neurotrophic factor, whereas Bax, Bad and caspase-9/-3 levels were significantly increased, both in terms of protein and mRNA levels in postmortem brains of BD patients. A recent clinical study also showed an increase in the Bax/Bcl-2 ratio and increased cytochrome *c* release and caspase-3 activity in manic and depressed patients compared to healthy subjects.^15^ Similarly, lymphocytes from patients with BD present decreased expression of the HSP70, an anti-apoptotic factor, as well as a decrease in Bax levels in the cytosolic fraction, suggesting Bax activation and translocation to the mitochondria to induce apoptosis.^16^ Moreover, a study published by Fries *et al.*^17^ showed an increased percentage of early apoptotic cells in euthymic patients with BD when compared to controls, but no differences in overall cell viability, necrosis or late apoptosis, suggesting potential plasticity and an intervention opportunity.

Mitochondria are organelles responsible for multiple cellular functions, including ATP production, metabolism of reactive oxygen species and calcium homeostasis.^18, 19^ Moreover, they represent a convergence point for death signals activated by both intracellular and extracellular stimuli, leading to the release of apoptogenic factors, such as cytochrome *c*, apoptosis-inducing factor, and SMAC/Diablo.^20^ In addition, recent studies indicate that mitochondrial fission/fusion machinery actively participates in the process of apoptosis, since dysregulation of mitochondrial dynamics leads to mitochondrial fragmentation.^21, 22^ However, the specific processes of apoptosis and mitochondrial dynamics in BD have not been fully elucidated. To test the above hypothesis, we measured the protein levels of several pro- (Bax, Bad, Bak, Smac and active caspase-3) and anti-apoptotic factors (Bcl-xL, Bcl-xL/Bak dimer, survivin and Mcl-1), the mRNA and protein expression levels of mitochondrial fission/fusion proteins (Mfn2, Opa1 and Fis1), and the mitochondrial content in peripheral blood mononuclear cells (PBMCs) from BD patients and healthy controls.

## Materials and methods

### Subjects

This study was carried out in accordance with the principles of the Declaration of Helsinki with approval from the Institutional Review Board of the University of Texas Health Science Center at Houston, and written informed consent was obtained from all research participants. Sixteen participants with BD type I were recruited from the UTHealth Mood Disorders outpatient clinic, and 16 healthy controls were recruited from the local community and did not have a personal psychiatric disorder or family history of major psychiatry disorder or neurologic disorders in first-degree relatives. Specific inclusion criteria for the BD patients were as follows: (a) BD patients with diagnosis of BD I, based on DSM-IV criteria; (b) 18–65 years old; (c) BD patients at any current mood state at the time of the study; (d) BD patients preferably off pharmacological treatment at the time of study, but if not feasible, being on antidepressants and mood stabilizers (including anticonvulsants, typical and atypical antipsychotics and lithium will be allowed). The healthy controls were matched for age, gender, race/ethnic background and socioeconomic status. Subjects with current or past axis I DSM-V psychiatric disorders or first-degree relatives with any axis I psychiatric disorder will be excluded. All subjects completed the Mini-International Neuropsychiatric Interview (MINI) in order to confirm BD diagnosis in patients or to exclude a history of psychiatric disorders in controls.^23^ BD participants were also assessed using the Montgomery–Åsberg Depression Rating Scale (MADRS)^24^ and the Young Mania Rating Scale (YMRS)^25^ to index the severity of depressive and manic symptoms, respectively. Functioning was assessed with the Global Assessment of Functioning (GAF) Scale and Functioning Assessment Short Test (FAST)^26, 27^

### Processing whole-blood samples

Human blood samples were collected in heparin-coated collection tubes. Then, PBMCs were separated using LeucoPREP brand cell separation tubes (Becton Dickinson Labware, Lincoln Park, NJ, USA). PBMC cell pellets were mixed with RPMI-1640 medium containing 10% dimethyl sulfate and frozen overnight in a Mr. Frosty container with 2-propanol (#5100–0001, Nalgene, Rochester, NY, USA) at −80 °C following an appropriate post-processing delay.

### Quantification of the intrinsic apoptotic pathway

The intrinsic apoptotic pathway was assayed using multiplex fluorescent immunoassay kits (Bio-Plex Pro RBM apoptosis assays). The xMAP platform used here was based on the Rules-Based Medicine (RBM) fluorescent beads and antibody pairs. These are sensitive, specific and widely used reagents, sourced by numerous manufacturer's and the data collected using xMAP multiplex beads are widely reported in the literature in studies in which multiple proteins are assayed simultaneously. Total cellular extracts from the PBMC cells were prepared by lysing the cells in lysate dilution buffer according to the manufacturer’s instructions, followed by centrifugation at 4 °C for 10 min at 10 000 *g*.

The assays were conducted in 96-well polystyrene, round-bottom microplates. Initially, blocker (10 μl) was added to all wells of the plate. Next, 30 μl of the standard, control or total extracts were added to each well, as indicated. A 10 μl aliquot of the working bead mixture was transferred into the wells. The plate was incubated on a plate shaker (850 r.p.m.) in the dark at RT for 60 min. The plate was then placed in the magnetic separator and incubated for separation for 60 s. The supernatant was carefully removed from each well by manual inversion. Beads were washed 3 times by adding 100 μl of assay buffer into each well to ensure the absence of any undesirable or non-specifically bound antibodies. After this protocol, 40 μl of a detection antibody were added to each well. Incubation was again conducted in darkness and at RT on a plate shaker (850 r.p.m.) for 60 min. Finally, 20 μl of streptavidin-PE was added to each well. The plate was incubated on a plate shaker (850 r.p.m.) in the dark at RT for 30 min. The supernatant was carefully removed after magnetic separation of the beads by manual inversion, and washing was performed as previously described. Assay buffer (100 μl) was added into each well, and the plate was placed onto a plate shaker for approximately 30 s in order to achieve gentle agitation of the beads. Samples were run in duplicate using a Bioplex system (Bio-Plex 200 Systems, BioRad, Hercules, CA, USA) and data analysis was conducted in Bio-Plex Manager 4.0 using a 5-parameter logistic regression model.

### Gene expression

PBMCs from each subject were used for total RNA isolation using the RNeasy Plus Mini Kit (Qiagen, Germantown, MD, USA), according to the manufacturer’s instructions. After quantification on NanoDrop (Uniscience, Hialeah, FL, USA), the RNA samples were converted to complementary DNA (cDNA) with the High-Capacity cDNA Reverse Transcription Kit (Applied Biosystems, Foster City, CA, USA). The cDNA synthesis was performed in a final volume of 10 μl with 2 μl 10x RT buffer, 0.8 μl 25 × dNMT Mix, 2 μl 10 × RT random primers and 1 μl MultiScribe reverse-transcriptase (50 units per ml), and the reactions were incubated for 10 min at 25 °C, 2 h at 37 °C, and 5 s at 85 °C. Real-time quantitative reverse-transcriptase PCR (qRT–PCR) reactions were performed for the assessment of *Mfn1*, *Mfn2*, *Opa1*, *Drp1* and *Fis1* gene expression using specific TaqMan FAM/MGB assays (Applied Biosystems, ID assay Hs00966851_m1 for *Mfn1*, ID assay Hs00208382_m1 for *Mfn2*, ID assay Hs01047018_m1 for *Opa1*, ID assay Hs01552605_m1 for *Drp1*, and Hs00211420_m1 for *Fis1*). GAPDH (Applied Biosystems, VIC/MGB assay ID Hs99999905_m1) was used as the endogenous control. Reactions were performed in triplicate in a final volume of 12 μl containing 6 μl 2 × TaqMan Gene Expression Master Mix, 0.6 μl 20 × TaqMan Gene Expression Assay, 0.6 μl 20 × TaqMan Endogenous Control, 3.8 μl of water, and 1 μl of cDNA solution, and were run in a QuantStudio 7 Flex Real-Time PCR System (ThermoFischer). Gene expression levels were analyzed by the ddCt method.^28^

### Enzyme-linked immunosorbent assay

Proteins were extracted from PBMCs using 1 × Cell Lysis Buffer (Bio-Plex Cell Lysis Kit, Bio-Rad, Hercules, CA, USA) containing 500 mM PMSF (Sigma-Aldrich, St. Louis, MO, USA). Insoluble debris were removed by centrifugation at 4500 *g* for 20 min at 4 ºC. Total protein concentration was determined using a BCA assay (Thermo Scientific Pierce). Afterwards, Mfn1, Mfn2, Opa1, Fis1 and Drp1 levels were determined using sandwich-ELISA assays (MyBioSource, San Diego, CA, USA) according to the manufacturer's instructions. The standard curve demonstrated a direct relationship between optical density (OD) and protein concentration.

### Citrate synthase activity

Citrate synthase activity was assayed according to the method described by Srere,^29^ with a reaction mixture containing 100 mM Tris, pH 8.0, 0.1 mm acetyl CoA, 0.1 mm 5,5′-di-thiobis-(2-nitrobenzoic acid), 0.1% triton X-100, and 2–4 μg supernatant protein. The enzymatic reaction was initiated with 0.2 μm oxaloacetate and monitored at 412 nm for 3 min at 25 °C.

### mtDNA copy number

Real-time quantitative (PCRs) were performed to measure the amount of mitochondrial DNA relative to a single-copy gene (beta-hemoglobin) with a modified protocol from Tyrka and collaborators.^30^ Reactions included 25 ng genomic DNA, 300 nmol l^−1^ of each primer, and 1 × Sybr Select Master Mix (Life Technologies, Carlsbad, CA, USA) in a final volume of 10 μl. Primer sequences and PCR cycling conditions for both mtDNA and beta-hemoglobin have been previously reported.^30^ Reactions were carried out in 96-well plates and data were acquired in a QuantStudio 7 Flex Real-Time PCR System (Life Technologies). mtDNA copy number for each sample was determined by relative quantification based on a 5-point standard curve performed with a serial dilution (1:2) of a calibrator sample ranging from 1 to 0.0625 ng DNA. All samples were analyzed in triplicate. The relative amount of mtDNA was finally divided by the relative amount of the beta-hemoglobin gene to obtain an index of mitochondrial DNA copy number.

### Statistical analysis

Statistical analyses were performed using Statistical Package for the Social Sciences, v.23.0 (SPSS, Armonk, NY, USA). Descriptive statistics were used to report demographic and clinical characteristics of the sample. Normality of data distribution was assessed using the Shapiro-Wilk test and histogram visualization. Since the variables being analyzed did not follow a normal distribution, the nonparametric Mann–Whitney U-test was used to test significant differences between the groups. Categorical variables were compared using chi-square or Fisher’s exact tests. Correlation analyses were performed using Spearman's rank correlation test. Data are presented as median and interquartile range (IQR). Significance was set at *P*<0.05. Analyses of covariance were used to adjust for possible confounders. We considered all variables associated with diagnostic group and markers with *P*<0.20 to be possible confounding factors.^31^

## Results

Characteristics of BD participants and healthy controls are shown in Table 1. The intrinsic apoptotic pathway and mitochondrial dynamics were evaluated in 16 participants with BD and 16 healthy controls. Notably, no significant differences in sociodemographic factors (ethnicity, socioeconomic status, age, gender, body mass index, education and smoking status) emerged between groups. The mean YMRS score of the BD group was 7.75±6.97, and their mean MADRS score was 13.81±10.94. Moreover, a significant difference in functional status, assessed by GAF (*P*<0.001) and FAST (*P*<0.001), was found between healthy controls and patients with BD. All patients were on treatment with various psychiatric medications at conventional doses at the time of the study, including antipsychotics, anticonvulsants, mood stabilizers and antidepressants. Some patients received additional benzodiazepines and stimulants.

**Figure 1:**
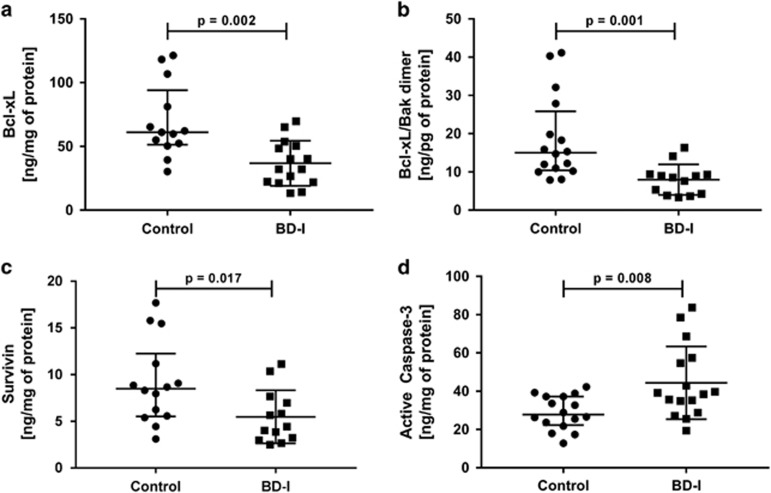
Alteration of anti- and pro-apoptotic proteins in peripheral blood mononuclear cells (PBMCs) from healthy controls and patients with Bipolar Disorder type I (BD-I). (**a**) Protein levels of Bcl-xL, (**b**) Protein levels of Bcl-xL/Bak dimer, (**c**) Protein levels of survivin and (**d**) Protein levels of active caspase-3. Data were presented as median and interquartile range (IQR). Differences between 2 groups were compared using the Mann–Whitney U-test. Different from the control group; *P<0.05.

The role of mitochondrial pathway-mediated apoptosis markers in BD was investigated by analyzing the protein levels of bcl-2 family proteins, survivin, smac, and lamin B using multiplex fluorescent immunoassay kits. The levels of the anti-apoptotic proteins Bcl-xL (*P*=0.002), Bcl-xL/Bak dimer (*P*=0.001) and survivin (*P*=0.017) were significantly lower in PBMCs from the BD group compared to lymphocytes from the healthy control group. However, the levels of active caspase-3 (*P*=0.008) were significantly higher in lymphocytes from the BD group when compared with healthy controls (Figure 1). In contrast, no differences in the protein levels of the pro-apoptotic factors Bad, Bax, Bad and Smac, or in the anti-apoptotic proteins Mcl-1 and Mcl-1/Bak dimer were found in lymphocytes from the BD group when compared with healthy controls (Supplementary Figure 1S).

**Figure 2:**
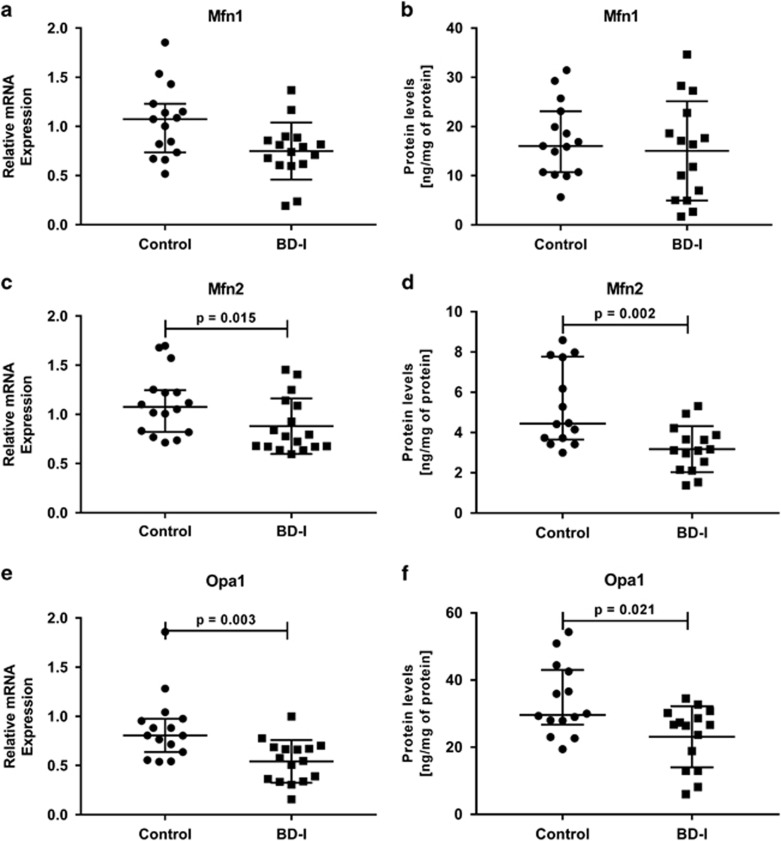
Alteration of mitochondrial fusion proteins in peripheral blood mononuclear cells (PBMCs) from healthy controls and patients with Bipolar Disorder type I (BD-I). (**a**) Relative mRNA expression of *Mfn1*, (**b**) Protein levels of Mfn1 (**c**) Relative mRNA expression of *Mfn2*, (**d**) Protein levels of Mfn2, (**e**) Relative mRNA expression of *Opa1*, (**f**) Protein levels of Opa1. Data were presented as median and interquartile range (IQR). Differences between 2 groups were compared using the Mann–Whitney U-test. Different from the control group; *P<0.05.

**Figure 3:**
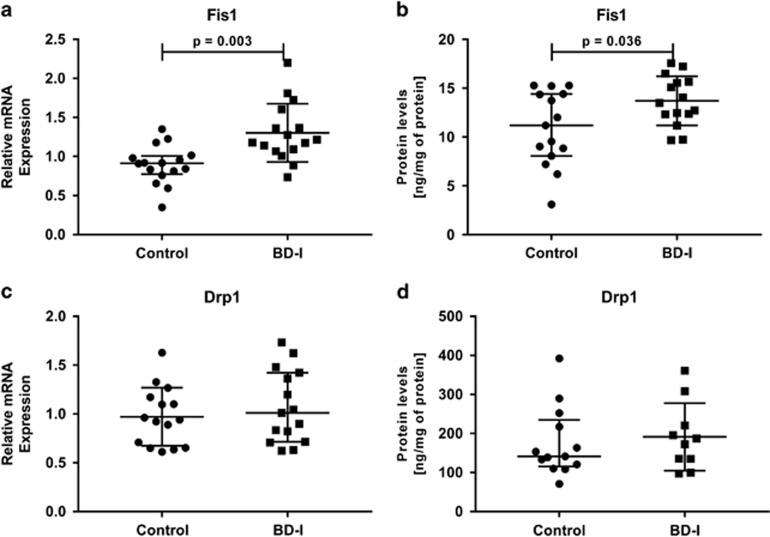
Alteration of mitochondrial fission proteins in peripheral blood mononuclear cells (PBMCs) from healthy controls and patients with Bipolar Disorder type I (BD-I). (**a**) Relative mRNA expression of *Fis1*, (**b**) Protein levels of Fis1, (**c**) Relative mRNA expression of *Drp1*, (**d**) Protein levels of Drp1. Data were presented as median and interquartile range (IQR). Differences between 2 groups were compared using the Mann–Whitney *U-*test. Different from the control group; *P<0.05.

To evaluate the mitochondrial dynamics of fusion and fission, we analyzed the mRNA expression and protein levels of Mfn1, Mfn2, Opa1, Drp1 and Fis1. We found that the gene expression of the mitochondrial fusion-related proteins *Mfn2* (*P*=0.015) and *Opa1* (*P*=0.003) was downregulated in the BD group compared to healthy controls (Figures 2c and e, respectively). On the contrary, the gene expression levels of mitochondrial fission-related protein *Fis1* (*P*=0.003) were significantly higher in the BD group compared to healthy controls (Figure 3a). Moreover, there were no significant differences in the gene expression levels of *Mfn1* and *Drp1* in PBMCs between healthy controls or BD subjects (Figures 2a and 3c, respectively). The overexpression or reduced expression of mitochondrial fission/fusion proteins was confirmed by ELISA analysis. As expected, the results revealed that the protein levels of Mfn2 (*P*=0.002) and Opa1 (*P*=0.021) were significantly decreased in BD patients compared with the controls (Figures 2d and f, respectively), whereas the protein levels of Fis1 (*P*=0.036) were significantly increased in lymphocytes from the BD group when compared with healthy controls (Figure 3b).

**Figure 4:**
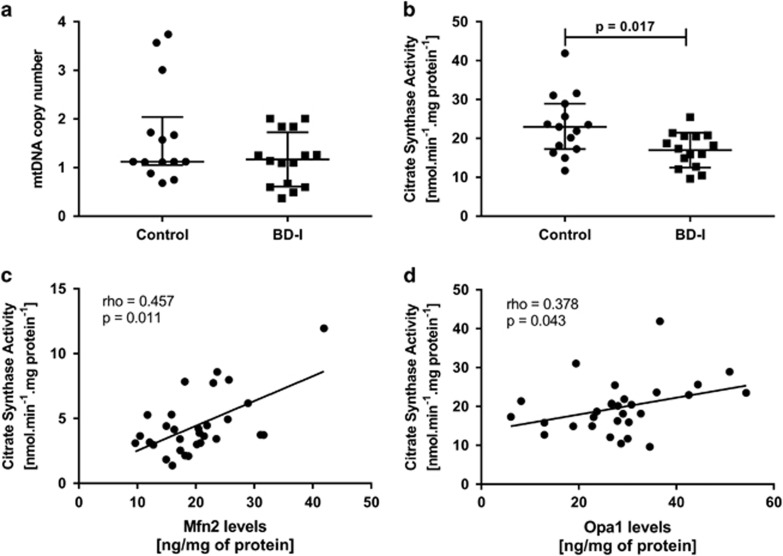
Mitochondrial mass in in peripheral blood mononuclear cells (PBMCs) from healthy controls and patients with Bipolar Disorder type I (BD-I). (**a**) Distribution of relative mtDNA copy number. (**b**) Citrate synthase activity. (**c**) Correlation coefficient (Pearson's correlation coefficient) between citrate synthase activity and protein levels of Mfn2. (**d**) Correlation coefficient (Spearman's rank correlation test) between citrate synthase activity and protein levels of Opa1. Data were presented as median and interquartile range (IQR). Differences between 2 groups were compared using the Mann–Whitney *U-*test. Different from the control group; *P<0.05.

In addition, to establish whether the changes observed in the fusion and fission process occurred in conjunction with alterations in mitochondrial mass, we evaluated mitochondrial content by quantifying mtDNA copy number. We observed no significant difference in relative mtDNA copy number in the BD group (Figures 4a, *P*=0.315). To verify whether the mtDNA content in BD was associated with mitochondrial mass, we performed citrate synthase activity assay, an enzymatic marker of mitochondrial mass. We observed a significant decrease in citrate synthase activity in lymphocytes from the BD group when compared with healthy controls (Figures 4b, *P*=0.017). Moreover, we also observed positive correlations between mtDNA content and citrate synthase activity (rho=0.483, *P*=0.008), suggesting a reduced mitochondrial mass in BD patients. Next, we analyzed the relationship between fusion/fission proteins with citrate activity. As seen in Figure 4c, Mfn2 levels were positively correlated with citrate synthase activity (rho=0.457, *P*=0.011). The present results also show a statistically significant correlation between Opa1 protein levels and citrate synthase activity (rho=0.378, *P*=0.043; Figure 4d). No correlations were found between Fis1 protein levels, citrate synthase activity and mtDNA (data not shown).

**Figure 5:**
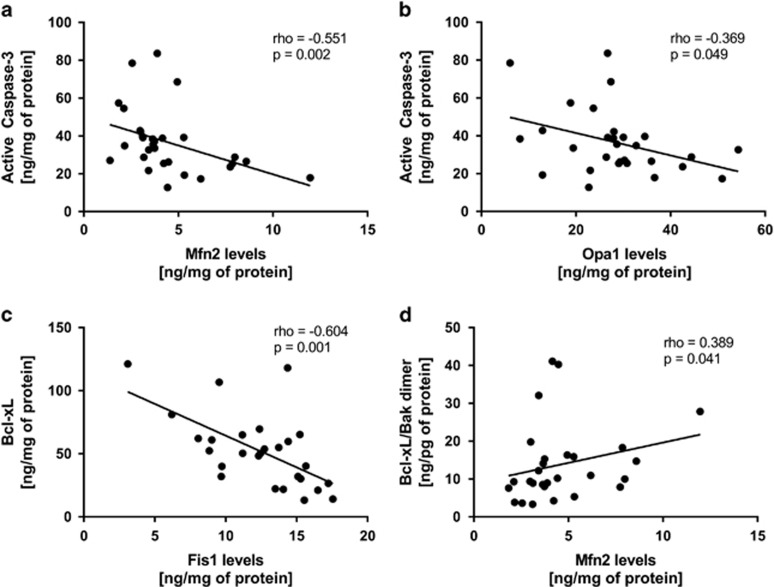
Correlation coefficient of active caspase-3, Bcl-xL and Bcl-xL/Bak dimer protein levels with Mfn2, Opa1 and Fis1 in peripheral blood mononuclear cells (PBMCs) from healthy controls and patients with Bipolar Disorder type I (BD-I). Results were assessed using the Spearman's rank correlation test.

Since Bcl-2 family members play an active role in the regulation of mitochondrial dynamics, we analyzed the relationship between apoptosis and mitochondrial fission/fusion proteins. The present results show that active caspase-3 was negatively correlated with Mfn2 levels (rho=−0.551, *P*=0.002) and Opa1 levels (rho=−0.369, *P*=0.049). Also, a significant negative correlation was found between Bcl-xL and Fis1 levels (rho=−0.604, *P*=0.001), while a positive correlation was found between Bcl-xL/Bak dimer and Mfn2 levels (rho=0.389, *P*=0.041; Figure 5). No correlations were found between Mfn2 protein levels and survivin protein levels (rho=0.065, *P*=0.748). However, we found a significant positive correlation between *Mfn2* mRNA levels and survivin protein levels (rho=0.432, *P*=0.024), as well as a positive correlation between *Mfn2* mRNA levels and Mfn2 protein levels (rho=0.420, *P*=0.021).

**Figure 6:**
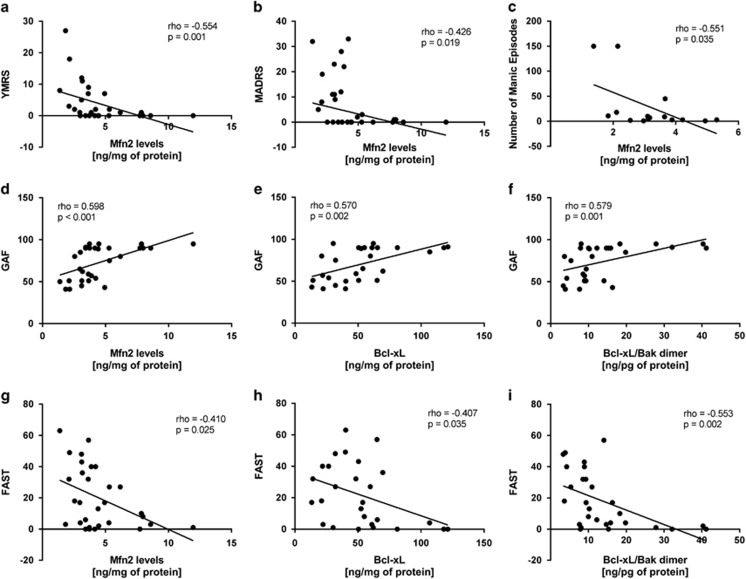
Correlation coefficient of Young Mania Rating Scale (YMRS) score (**a**), Montgomery-Asberg Depression Scale (MADRS) score (**b**), number of manic episodes (**c**), Functional Assessment Screening Tool (FAST) scale (**d**) and Global Assessment of Functioning (GAF) scale (**e**) with Mfn2 protein levels in peripheral blood mononuclear cells (PBMCs) from healthy controls and patients with Bipolar Disorder type I (BD-I). Results were assessed using the Spearman's rank correlation test.

Finally, in an attempt to explore the potential clinical correlates of our findings, we tested possible correlations between psychiatric and demographic characteristics and biochemical data. Our results showed no significant differences in the protein levels of apoptotic factors and mitochondrial fission/fusion proteins in patients under treatment with lithium and antipsychotics (data not shown). Moreover, no associations were found between cellular parameters and age, gender, ethnicity, education, body mass index, smoking, number of depressive episodes or number of hospitalizations (*P*>0.05 for all analyses). However, we found a negative correlation between Mfn2 levels and YMRS (rho=−0.554, *P*=0.001, Figure 6a) and MADRS (rho=−0.426, *P*=0.019, Figure 6b) scores, as well as with the number of manic episodes (rho=−0.551, *P*=0.035, Figure 6c). Moreover, we also performed an additional analysis to test the hypothesis that the alterations in anti-apoptotic factors and mitochondrial fission/fusion proteins correlate with the functional status. The results of Spearman’s rank correlation showed a positive correlation between the GAF scores and Mfn2 (rho=0.598, *P*<0.001, Figure 6d), as well as with the anti-apoptotic factors Bcl-xL (rho=0.570, *P*=0.002, Figure 6e) and Bcl-xL/Bak dimer (rho=0.579, *P*=0.001, Figure 6f). Similarly, FAST scores were negatively correlated with Mfn2 (rho=−0.401, *P*=0.025, Figure 6g), Bcl-xL (rho=−0.407, *P*=0.035, Figure 6h) and Bcl-xL/Bak dimer (rho=−0.553, *P*=0.002, Figure 6i).

## Discussion

Brain imaging studies over the last two decades have confirmed the presence of volumetric abnormalities in patients with BD compared with healthy control subjects, including reductions in gray matter density in the hippocampus, amygdala, frontal cortex, superior temporal gyrus, cerebellum, and ventricular systems.^32, 33, 34, 35, 36, 37^ Previous studies have also reported that the number and size of glia and neurons are reduced in some of these brain areas,^38, 39^ suggesting the involvement of apoptosis in the pathophysiology of BD. However, the specific processes of apoptosis in BD have not been fully elucidated. In this study, we have investigated the levels of several survival-related and pro-apoptotic proteins in PBMCs of BD participants, and we observed significant alterations compared to healthy controls, including marked decreases in the levels of Bcl-xL, survivin and Bcl-xL/Bak dimers and an increase in the caspase-3 protein levels. Our results are thus in agreement with previous studies showing changes in apoptosis in PBMCs of patients with BD and in postmortem prefrontal cortex from BD patients.^12, 15, 40^

Mitochondria play a key role in the regulation of apoptosis.^41^ Specifically, the release of various pro-apoptotic proteins that are normally present in the intermembrane space of these organelles, such as the cytochrome *c*, is observed during the early stages of apoptotic cell death.^14, 42^ Anti-apoptotic members of the Bcl-2 family, such as Bcl-2 or Bcl-xL, inhibit Bax or Bak activation, as members of the Bcl-xL protein family contain BH1 and BH2 domains which are required for heterodimerization with Bak and the inhibition of cell apoptosis.^43^ Consistent with this, we observed a decrease in Bcl-xL and Bcl-xL/Bak heterodimers, suggesting that Bcl-xL cannot protect against apoptosis by inhibiting Bak oligomerization. This can lead to the formation of mitochondrial pores and allows the release of intermembrane space proteins that can either act as cofactors for the assembly of the Apaf-1/caspase-9 apoptosome or promote other downstream events in apoptosis, ultimately leading to caspase-3 activation and subsequent cell death. Moreover, to our knowledge, this is the first study to show significantly lower levels of survivin in PBMCs of BD patients compared to healthy controls. Survivin, a member of the family of inhibitors of apoptosis proteins (IAPs), is a bifunctional protein that regulates cell division and suppresses apoptosis.^44, 45^ It represents a chromosomal passenger protein that binds to caspases, both the effectors caspase-3 and caspase-7 and the initiator caspase-9,^46, 47, 48^ thus protecting cells from apoptosis. Iscru *et al.* (2013) reported that survivin was also involved in LTP in the hippocampus, suggesting that survivin is involved in hippocampal synaptic plasticity either by compromising hippocampal neurogenesis or by direct interactions with signaling cascades such as NF-kappa B that are involved in hippocampal synaptic plasticity and learning. Thus, a marked decrease of survivin in PBMCs of BD patients would change the balance between the levels of active caspases and IAPs, allowing the active caspases to execute apoptosis. Finally, if taken as a proxy of the brain, the decrease detected in our sample could lead to neuronal loss and impaired synaptic plasticity in BD patients. Corroborating this hypothesis, our results showed higher levels of active caspase-3.

Of note, the interplay between the Bcl-2 family and mitochondria is not limited to the intrinsic pathway. Bcl-2 family members play an active role in the regulation of mitochondrial dynamics, mitochondrial respiration, metabolism, and apoptotic resistance.^49, 50, 51, 52^ In this study, real-time PCR and ELISA were used to measure the expression and protein levels of mitochondrial fusion/fission proteins in PBMCs from BD patients. Compared to healthy controls, BD patients showed downregulation of *Mfn2* and *Opa1* and upregulation of *Fis1* both in terms of gene expression and protein levels. Having analyzed mtDNA, we also observed a trend for a decrease in copy number in the BD group. Our results are in accordance with a previous study in leukocyte mtDNA, which showed a slight decrease in baseline mtDNA content in type I BD compared to controls. Of note, our results show that patients present a decrease in the activity of citrate synthase, a marker of mitochondrial content, which were positively correlated with mtDNA. Moreover, decreased Mfn2 protein levels were correlated with the activity of citrate synthase and mtDNA, suggesting that Mfn2 is essential for the regulation of mitochondrial morphology by tipping the balance towards mitochondrial fragmentation. Our results are supported by a study published by Cataldo *et al.*^53^ demonstrating that prefrontal cortex neurons of postmortem brain from patients with BD and peripheral cells from BD patients display morphological abnormalities (more mitochondria of smaller size) and an abnormal pattern of clumping and marginalization in the intracellular distribution of mitochondria. Taken together, these results suggest that these mitochondrial structural abnormalities may represent an attempt to overcome a reduced mitochondrial network connectivity, as determined by the balance between fusion and fission processes.

**Figure 7:**
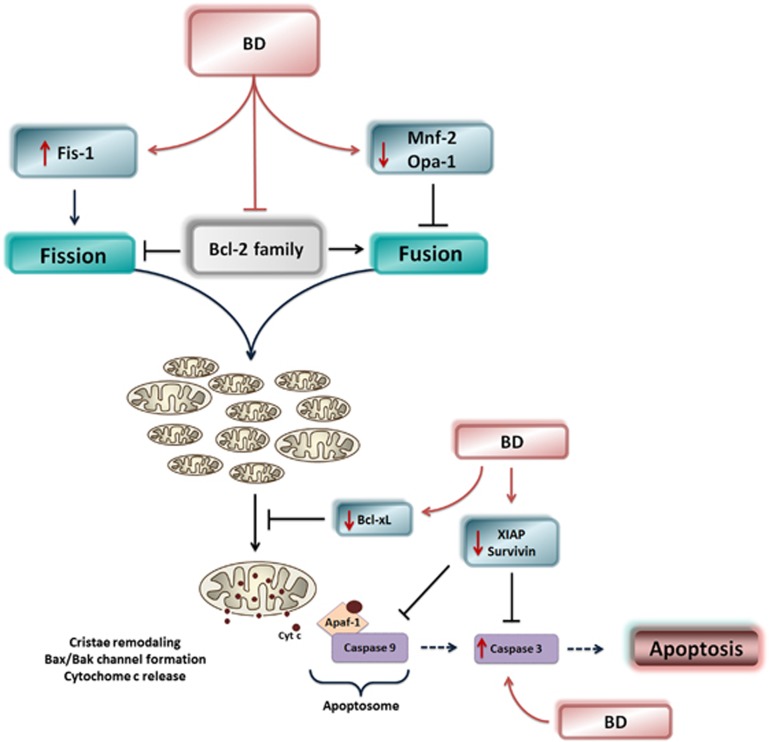
Schematic diagram illustrating the disruption of the balance between mitochondrial fusion and fission in bipolar disorder (BD) patients, and also the modulation of Bcl-2 family. This imbalance in mitochondrial fission and fusion towards fission causes mitochondrial fragmentation and may directly drive the cell into apoptosis, leading to the release of pro-apoptotic proteins, cristae remodeling, and activation of caspase-3. Moreover, members of the Bcl-2 family may participate in the regulation of mitochondrial fission/fusion dynamics in BD patients.

Several studies have demonstrated that alterations in proteins involved in mitochondrial fusion and fission lead to altered mitochondrial shape, loss of mtDNA, decrease in mitochondrial respiration, increased oxidative stress and apoptotic cell death.^54, 55, 56, 57^ Mfn2 expression controls the expression of oxidative phosphorylation, and the inhibition of Mfn2 expression is associated with a reduced mitochondrial membrane potential, impaired glucose oxidation and a downregulation of oxidative phosphorylation subunits, all of which markedly alters the extent of the mitochondrial network.^58, 59^ Mfn2 deficiency also increases the mitochondrial Bax/Bcl-2 ratio, indicating that Mfn2 deficiency potentially induces apoptosis through the Bcl-2 and Bax pathway.^60^ Depletion of Opa1 or mitofusins results in poor cell growth and enhances susceptibility to apoptotic stimuli^55, 61, 62^ because loss of the inner mitochondrial membrane protein Opa1 leads to drastic reorganization of mitochondrial cristae, fragmentation of mitochondria, efflux of cytochrome *c*, and apoptosis.^61^ Moreover, excessive mitochondrial fission mediated by Drp1 and Fis1 produces dysfunctional mitochondrial fragments that show increased production of reactive oxygen species and are also more sensitive to mitochondrial permeability transition pore opening and Bax/Bak activation.^49, 63^ In this study, we showed that patients with BD demonstrated increased levels of active caspase-3, which were shown to be negatively correlated with Mfn2 and Opa1. Thus, the downregulation of Mfn2 and Opa1 and overexpression of Fis1 observed in BD patients can induce mitochondrial fission and apoptosis because mitochondrial fragmentation induced by Fis1 is followed by cytochrome *c* release, caspase activation and cell death.^64^ Taken together, we suggest that an imbalance in mitochondrial fission and fusion towards fission causes mitochondrial fragmentation and may drive the cell into apoptosis, leading to the release of pro-apoptotic proteins, cristae remodeling, and activation of caspase-3. Moreover, Bcl-2 family members may participate in the regulation of mitochondrial fission/fusion dynamics in BD patients because Bcl-2 levels were markedly decreased in BD patients,^12, 15^ and this protein has an antagonistic effect on mitochondrial fission machinery mediated by the mutually exclusive interactions of Bcl-2 and Drp1 with Fis1.^65^ In support of this hypothesis, we found that the levels of Bcl-xL and Bcl-xL/Bak dimer, members of the Bcl-2 family, were correlated with Mfn2 and Fis1 levels, suggesting that Bcl-2 family can regulate mitochondrial fission/fusion dynamics in BD patients (Figure 7).

Several studies have shown parallel alterations in central nervous system and peripheral cells of patients with BD. In addition, BD patients show higher rates of medical comorbidities, including cardiovascular disease, diabetes mellitus and obesity, which led some investigators to conceptualize BD as a multi-systemic disease.^66, 67, 68^ In other words, peripherally-measured parameters can not only provide biomarkers of illness, but are also thought to influence behavior, cognitive and functional parameters. To corroborate this hypothesis, our results showed that individuals with worse functional status (as shown by lower GAF and higher FAST scores) showed significantly lower levels of fusions proteins Mfn2 and Opa1 and higher levels of Fis1. In the same line, we also found a negative correlation between functional status and Bcl-xL and Bcl-xL/Bak dimer. Accordingly, the changes in fission/fusion protein levels and anti-apoptotic factors observed in PBMCs from BD patients can support the relevance of the peripheral findings to mitochondrial abnormalities reported in the central nervous system of BD patients.

Our results should be interpreted in light of their limitations. First, our sample size was relatively small and this may limit the interpretation of our results. Second, it cannot be excluded that long-term treatment with psychiatric medications may affect the mitochondrial network. Third, our study was limited to mitochondrial analyzes that could be performed on frozen PBMC samples, which precluded us from assessing of other relevant mitochondrial parameters, such as oxygen consumption. Thus, future studies are necessary to verify these findings in larger and fresh samples, allowing the specific preparation of tissues for the analysis of mitochondrial morphology. These studies will be able to evaluate the morphological consequences of the imbalance of mitochondrial dynamics in patients with BD.

In conclusion, our results demonstrate significant changes in the expression of mitochondrial fission and fusion proteins and survival-related proteins in PBMCs from BD. Our findings in PBMCs of patients with BD support a link between mitochondrial dysfunction and the pathophysiology of BD, and the fact that these changes are seen in peripheral PBMCs suggests that the pathophysiological processes active in the disorder are body-wide and implicate circulating factors.^69^ Finally, understanding the mitochondrial network dynamics in BD and its interconnection with cellular signaling pathways may contribute to a more comprehensive understanding of the biological processes in this disease and may ultimately lead to the development of new treatment strategies.^70^

## Figures and Tables

**Table 1:** Demographic characteristics of all subjects

	*Healthy subjects*	*Bipolar subjects*	P*-value*
*n*	16	16	
Mean age, years	33.75±8.85	35.18±8.86	0.416
Female gender, *n* (%)	8 (50%)	8 (50%)	0.638
Ethnicity (non-Hispanic)	14 (87.5%)	12 (75%)	0.327
Education (years)	14.69±1.662	13.56±1.861	0.677
Body mass index	27.43 (5.82)	28.22 (6.38)	0.717
Smoking	18.80%	37.50%	0.217
MADRS	0.19 (0.403)	13.81 (10.94)	<0.0001
YMRS	0.13 (0.342)	7.75 (6.97)	<0.0001
GAF	90.13 (3.934)	56.00 (11.95)	<0.0001
FAST	5.38 (6.985)	34.00 (16.215	<0.0001
Number of manic episodes	NA	27.38 (49.10)	
Number of hypomanic episodes	NA	44.75 (41.02)	
Number of depressive episodes	NA	29.80 (55.56)	
Number of mixed episodes	NA	38.00 (50.56)	
Number of hospitalizations	NA	2.63 (3.70)	
Illness duration	NA	17.43 (7.78)	
Age at first mania episode	NA	19.11 (6.60)	
Age at first depressive episode	NA	15.31 (6.43)	
*Psychiatric medications (%)*			
Lithium	NA	25.00%	
Antidepressants	NA	31.30%	
Antipsychotics	NA	43.80%	
Anticonvulsant	NA	37.50%	
Benzodiazepines	NA	18.80%	
Stimulants	NA	6.30%	

Abbreviations: FAST, Functioning Assessment Short Test; GAF, Global Assessment of Functioning; MADRS, Montgomery–Åsberg Depression Rating Scale; NA, not applicable; YMRS, Young Mania Rating Scale.
