# Supplementary material for: Perturbations in the apoptotic pathway and mitochondrial network dynamics in peripheral blood mononuclear cells from bipolar disorder patients
Source: Transl Psychiatry. 2017 May 2;7(5):e1111–. doi: 10.1038/tp.2017.83 (PMC5534951; doi:10.1038/tp.2017.83)
Supplement: Supplementary Figure 1 [file tp201783x1.docx]

**Perturbations in the apoptotic pathway and mitochondrial network dynamics in peripheral blood mononuclear cells from Bipolar Disorder patients**

Giselli Scaini^1^, Gabriel R. Fries^1^, Samira S. Valvassori^2^, Cristian P. Zeni^3^, Giovana Zunta-Soares^3^, Michael Berk^4,5^, Jair C. Soares^3^, João Quevedo^1,2,3,6^

**
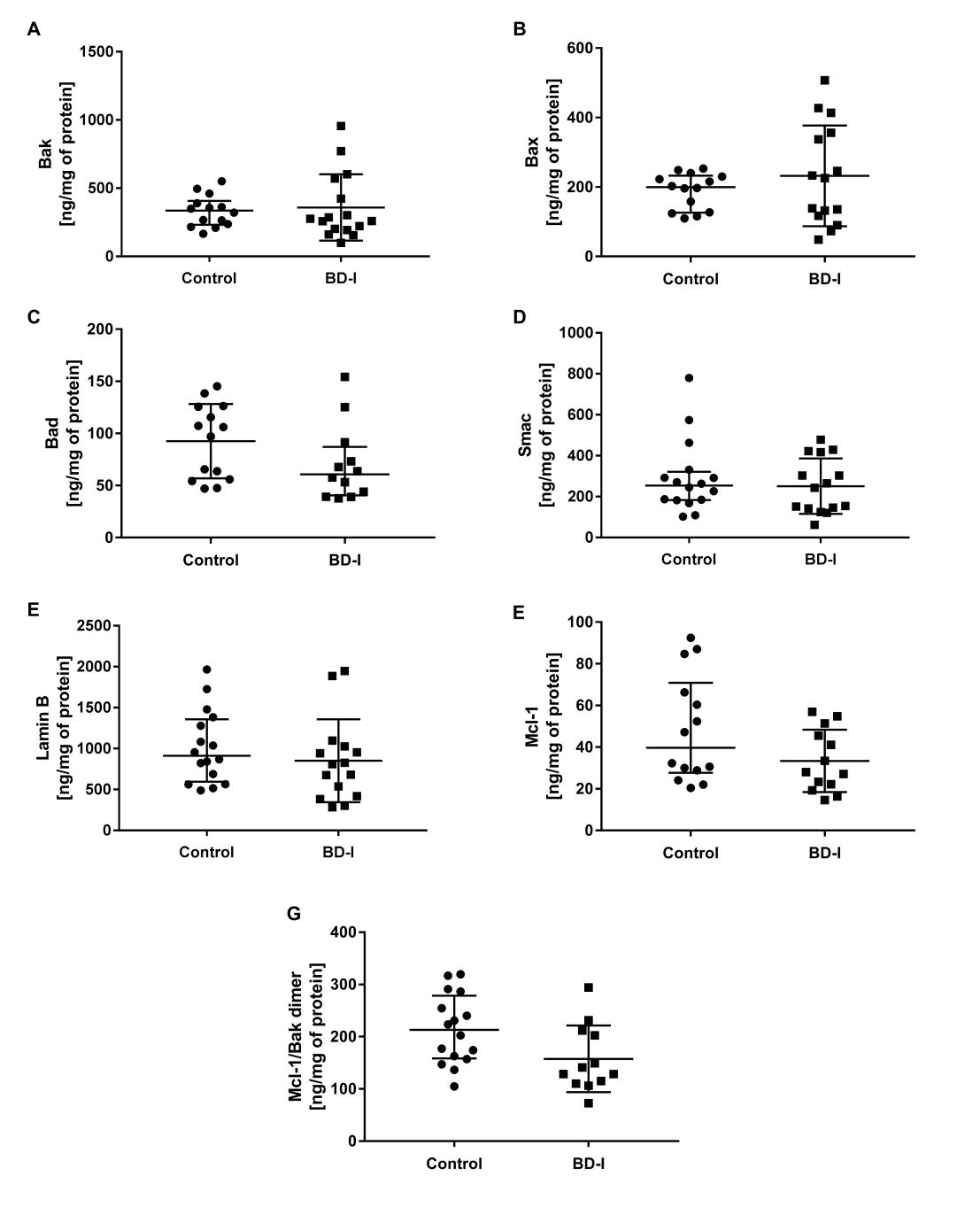
Supplementary Information**

**Figure 1: Alteration of pro- and anti-apoptotic proteins in peripheral blood mononuclear cells (PBMCs) from healthy controls and patients with Bipolar Disorder type I (BD-I).** (A) Protein levels of Bak, (B) Protein levels of Bax, (C) Protein levels of Bad, (D) Protein levels of Smac, (E) Protein levels of Lamin B, (F) Protein levels of Mcl-1, and (G) Protein levels of Mcl-1/Bak dimer. Data were presented as median and interquartile range (IQR). Differences between 2 groups were compared using the Mann-Whitney U test.
